# Supplementary material for: Comparative genomics provides new insights into the diversity, physiology, and sexuality of the only industrially exploited tremellomycete: Phaffia rhodozyma
Source: BMC Genomics. 2016 Nov 9;17:901. doi: 10.1186/s12864-016-3244-7 (PMC5103461; doi:10.1186/s12864-016-3244-7)
Supplement: Additional file 6: — List of orphan genes with links to PFAM (related to Additional file 1: Table S1). (ZIP 1428 kb) [file 12864_2016_3244_MOESM6_ESM.zip › PFAM_UNIQUE/G03761_P.html]

#                                                                            --- full sequence --- -------------- this domain -------------   hmm coord   ali coord   env coord
  
# target name        accession   tlen query name           accession   qlen   E-value  score  bias   #  of  c-Evalue  i-Evalue  score  bias  from    to  from    to  from    to  acc description of target
  
#------------------- ---------- ----- -------------------- ---------- ----- --------- ------ ----- --- --- --------- --------- ------ ----- ----- ----- ----- ----- ----- ----- ---- ---------------------
  
G03761\_P             -            613 DUF1675              PF07897.6    284       2.3    6.7  10.2   1   3       1.1   7.9e+02   -1.7   0.1   121   155   157   191   130   215 0.57 - Protein of unknown function (DUF1675)  
G03761\_P             -            613 DUF1675              PF07897.6    284       2.3    6.7  10.2   2   3    0.0037       2.7    6.4   4.1    82   228   297   442   288   445 0.76 - Protein of unknown function (DUF1675)  
G03761\_P             -            613 DUF1675              PF07897.6    284       2.3    6.7  10.2   3   3     0.066        49    2.3   0.0    12    53   508   549   505   560 0.86 - Protein of unknown function (DUF1675)
